# Non‐exposed endoscopic wall‐inversion surgery for an early gastric cancer arising from heterotopic submucosal gastric glands: A case report

**DOI:** 10.1002/deo2.70097

**Published:** 2025-03-11

**Authors:** Takeshi Abe, Yosuke Toya, Kyohei Sugai, Mizuki Komai, Shunichi Yanai, Haruka Nikai, Shigeaki Baba, Ryo Sugimoto, Naoki Yanagawa, Takayuki Matsumoto

**Affiliations:** ^1^ Division of Gastroenterology and Hepatology Department of Internal Medicine School of Medicine Iwate Medical University Iwate Japan; ^2^ Department of Surgery School of Medicine Iwate Medical University Iwate Japan; ^3^ Department of Molecular Diagnostic Pathology School of Medicine Iwate Medical University Iwate Japan

**Keywords:** early gastric cancer, heterotopic submucosal gastric glands, laparoscopic and endoscopic cooperative surgery, non‐exposed endoscopic wall‐inversion surgery, subepithelial lesion

## Abstract

A 74‐year‐old man, who was scheduled for surgery against the main duct‐type intraductal papillary mucinous neoplasm of the pancreas, was found to have a subepithelial lesion of the stomach under esophagogastroduodenoscopy. Endoscopic ultrasound‐guided fine needle aspiration for the gastric lesion revealed adenocarcinoma cells. We thus considered carcinomas arising from heterotopic submucosal gastric glands and metastases from the pancreatic lesion as differential diagnoses. We first non‐exposed endoscopic wall‐inversion surgery to the lesion as a total biopsy. The gastric lesion was diagnosed as early gastric cancer originating from heterotopic submucosal gastric glands. The patient subsequently underwent a pylorus‐preserving pancreatoduodenectomy for the intraductal papillary mucinous neoplasm. Our experience suggests non‐exposed endoscopic wall‐inversion surgery is a useful and minimally invasive option for the diagnosis and treatment of gastric submucosal lesions, which are presumed to be malignant in nature.

## INTRODUCTION

Laparoscopic and endoscopic cooperative surgery (LECS) is an accepted treatment of choice for gastrointestinal stromal tumors (GIST) to achieve minimal but complete resection.^1^ The initially reported form of LECS,^2^ known as “classical LECS,” involves opening the gastric wall during endoscopic dissection. This approach raises some concerns regarding intra‐abdominal spillage of gastric contents and tumor cells.

To address such issues, Goto et al.^3^ introduced a novel LECS, referred to as non‐exposed endoscopic wall‐inversion surgery (NEWS). NEWS is a full‐thickness resection technique, by which endoscopists and laparoscopists can refrain from any communication between the intra‐abdominal cavity and the intragastric spaces. NEWS is now regarded as a treatment option for early gastric cancer with little probability of lymph node metastasis because it allows for tumor resection without any possibility of tumor cell dissemination during the procedure.^4^

We herein report a case in which NEWS was useful for a definitive diagnosis of a subepithelial lesion (SEL) of the stomach.

## CASE REPORT

**FIGURE 1:**
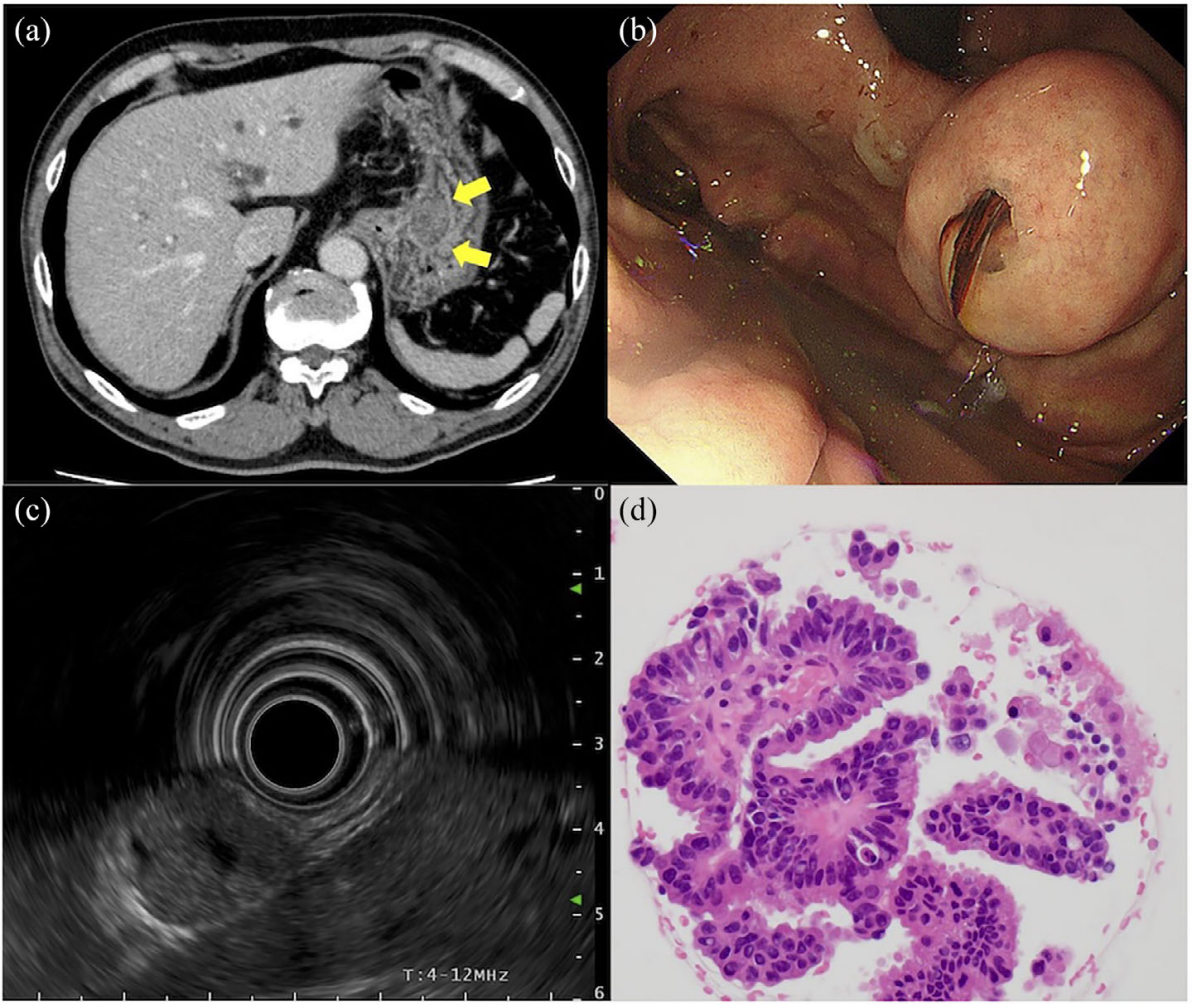
Computed tomography, endoscopic images, and a histological image of the gastric lesion from endoscopic ultrasound‐guided fine needle aspiration. (a) Computed tomography showed a tumor in the anterior wall of the gastric body (yellow arrows). (b) A 30 mm protruding lesion of a subepithelial lesion‐like appearance located on the anterior wall of the upper body. (c) Endoscopic ultrasonography showed that the lesion was a solid tumor originating from the fourth layer of the gastric wall with an internal hypoechoic area. (d) Endoscopic ultrasound‐guided fine needle aspiration detected adenocarcinoma cells.

A 74‐year‐old man was referred to our hospital for an evaluation of a pancreatic cyst. Computed tomography (CT) scan revealed a cystic lesion in the pancreatic head along with dilatation of the main pancreatic duct. The lesion was diagnosed as the main duct‐type intraductal papillary mucinous neoplasm (IPMN), which was considered to be an indication of pancreatoduodenectomy (PD). CT also revealed a gastric tumor, measuring approximately 30 mm, with a slightly heterogenous internal structure and well‐defined margins (Figure 1a)

Screening esophagogastroduodenoscopy for the preoperative evaluation revealed a protruding lesion of a SEL‐like appearance on the anterior wall of the upper gastric body. The lesion was measured approximately 30 mm in its largest size. There was a dimple in the center of the lesion, from which some bloody mucus was oozing (Figure 1b). Endoscopic ultrasonography (EUS; GF‐UE290; Olympus Co.) revealed that the lesion was a solid tumor originating from the fourth layer of the gastric wall with an internal hypoechoic area, which was suggestive of necrotic tissue (Figure 1c). Based on those endoscopic findings, we made a tentative diagnosis of GIST. We subsequently examined the lesion by EUS‐guided fine needle aspiration (EUS‐FNA), which detected adenocarcinoma cells (Figure 1d).

**FIGURE 2:**
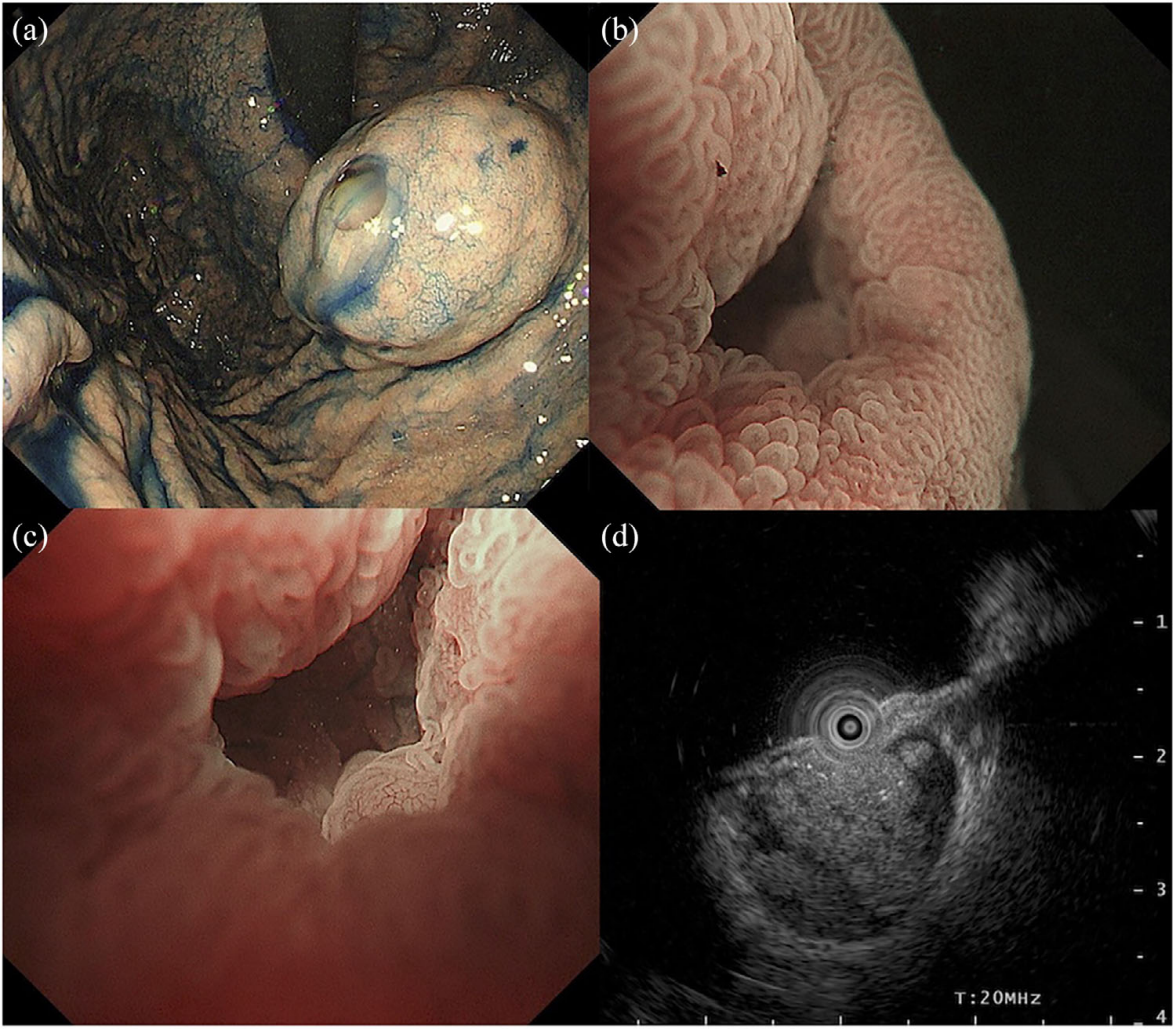
Detailed endoscopic images. (a) No obvious changes in the gross features of the lesion were observed. (b) Magnified endoscopy with narrow‐band imaging showed no evidence of intraepithelial neoplasm on the surface of the tumor. (c) Magnified endoscopy with narrow‐band imaging showed enlarged surface microstructures at the margins and villous structures in the center of the dimple. (d) Endoscopic ultrasonography with a miniature probe showed a lobulated solid component within the cystic lesion.

Because of the discrepancy between endoscopic and histologic findings, we repeated detailed esophagogastroduodenoscopy 1 month later, which showed no obvious changes in the gross features of the lesion (Figure 2a). In addition, magnifying endoscopy with narrow‐band imaging showed no evidence of intraepithelial neoplasm on the surface of the tumor (Figure 2b) except for enlarged surface microstructures at the margins and villous structures in the center of the dimple (Figure 2c). Furthermore, EUS with a miniature probe (UM‐3R; Olympus Co.) revealed a lobulated solid component floating within the cystic lesion (Figure 2d). While we could not reach a specific diagnosis, we considered SEL‐like gastric cancers, carcinomas arising from heterotopic submucosal gastric glands (HSG), and gastric metastases from intraductal papillary mucinous carcinoma to be candidate diagnoses. Due to the uncertainty in the invasion depth, the lesion was unlikely to be indicated for endoscopic removal.

Since gastrectomy plus PD seemed to be invasive for the patient, LECS, a minimally invasive treatment, was chosen for diagnostic therapy of gastric lesions. Because adenocarcinoma was suggested by the EUS‐FNA, we chose a non‐exposure technique for full‐thickness resection with a non‐exposure technique (CLEAN‐NET)^5^ instead of classical LECS. Because the tumor measured approximately 30 mm in diameter, which enables per‐oral removal of the resected specimen, we chose NEWS for this case after discussion with the surgeon.

**FIGURE 3:**
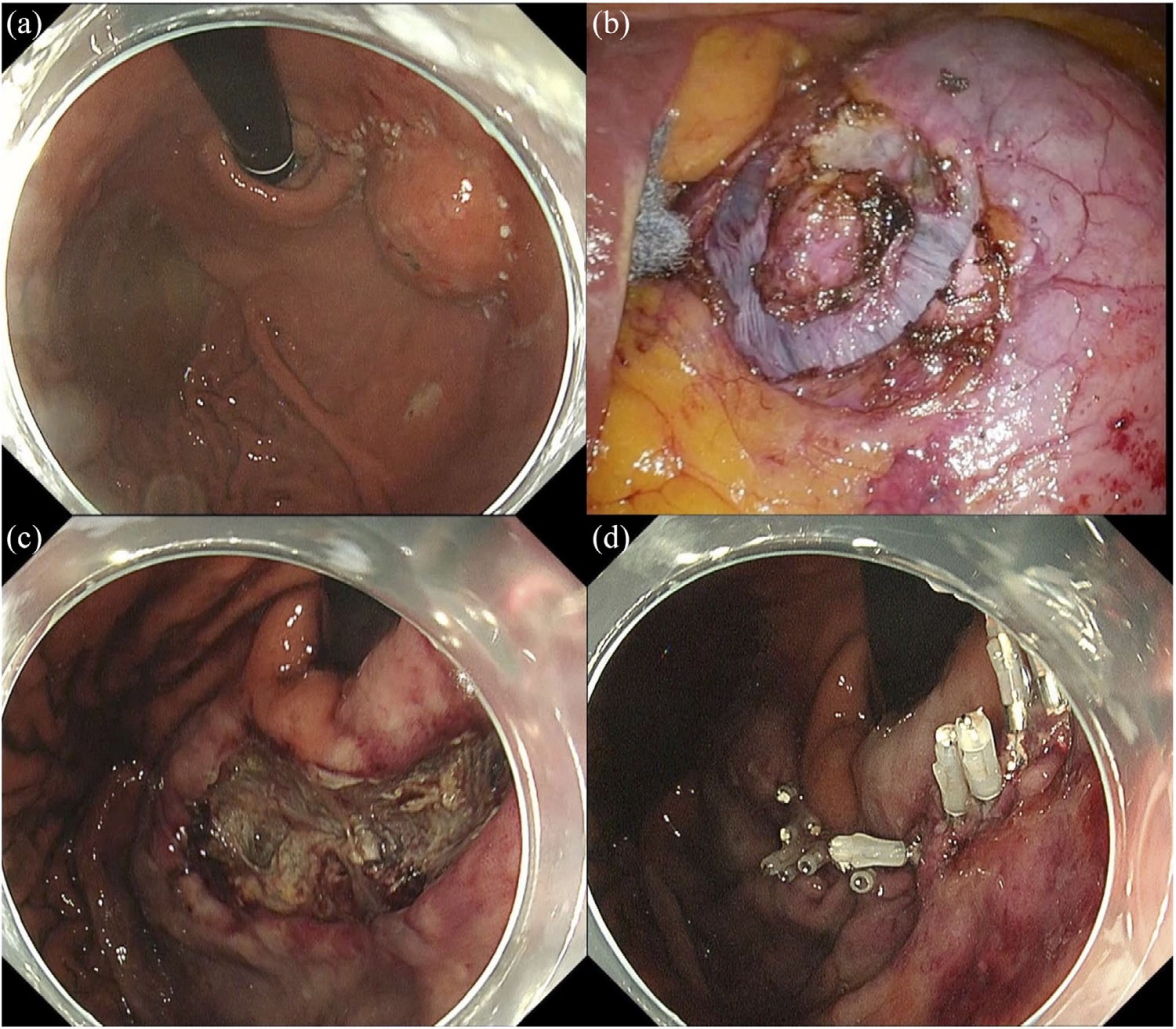
Non‐exposed endoscopic wall‐invasion technique. (a) The markings were placed to indicate the resection line. (b) The seromuscular layer around the tumor was incised laparoscopically. (c) The tumor was endoscopically removed with a submucosal dissection technique. (d) The mucosal defect was closed with laparoscopic suture and endoscopic clipping.

The NEWS procedure was performed using the following technique: (1) the margin of the tumor was identified and marked on both the mucosal and the serosal surface by coagulation to indicate the resection line (Figure 3a), (2) the seromuscular layer around the tumor was incised laparoscopically (Figure 3b), (3) the tumor was inverted into the intragastric cavity using a sponge spacer from the external wall of the stomach, (4) the serosal and muscular layers were sutured laparoscopically, (5) the tumor was endoscopically removed with a submucosal dissection technique and retrieved trans‐orally (Figure 3c), and (6) the mucosal defect was closed with endoscopic clipping (Figure 3d). The patient was discharged 10 days after NEWS without any complication.

**FIGURE 4:**
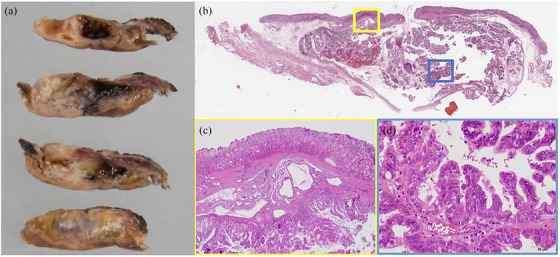
The resected specimen of the gastric lesion. (a) The tumor was 30 × 23 mm in size. Macroscopic findings showed cystic lesions with hemorrhage and mucus. (b) Hematoxylin‐stained loupe image showed that the tumor exhibited an inverted growth pattern with compression of the submucosa. (c) Heterotopic submucosal gastric glands (HSG) were observed around the tumor. (d) The dilated heterotopic submucosal gastric glands manifested a cyst‐like component, in which papillary proliferation of atypical cells exhibiting spindle‐shaped to round nuclei was evident.

Macroscopically, the tumor measured 30 × 23 mm in size. Macroscopic findings of the cut section contained cystic lesions with hemorrhage and mucus (Figure 4a). Pathologically, the tumor exhibited an inverted growth pattern surrounded by HSG, with a compression of the submucosa (Figure 4b). The dilated HGS manifested a cyst‐like component, around which papillary proliferation of atypical cells exhibiting spindle‐shaped to round nuclei was evident (Figure 4c,d). There was no tumor cell exposure on the mucosal surface. Desmin staining revealed discontinuous positive cells around the tumor and adjacent stroma, resembling mucosal lamina propria. The invasion depth was classified as pT1a. The final pathological diagnosis was well‐differentiated tubular adenocarcinoma with negative margins and no lymphovascular involvement. Immunohistochemical staining revealed that the tumor showed a gastric phenotype, with positive staining for MUC5AC and MUC6. In contrast, the epithelium of surrounding HSG was negative for MUC5AC and positive for MUC6.

Six months after NEWS, the patient underwent a pylorus‐preserving PD for IPMN. The histological diagnosis of the pancreatic lesion was determined to be IPMN of low‐grade dysplasia.

## DISCUSSION

In this report, we demonstrated gastric cancer arising from HSG, which could be diagnosed and successfully resected by NEWS. Gastric cancer arising from HGS is a condition difficult to diagnose by conventional and image‐enhanced endoscopy because it typically originates from gastric epithelium in the submucosa and manifests an SEL‐like configuration. With this regard, the lesion may be a candidate for removal by LECS. To the best of our knowledge, this is the first description of NEWS as a diagnostic and therapeutic approach for gastric cancer arising from HSG.

It has been reported that EUS is helpful for preoperative diagnosis of cancer arising from HGM because it can depict hypoechoic areas within the tumor, which are characteristic of HSG.^6^ However, Hagiwara et al. reported that EUS is not substantial because the procedure cannot distinguish cancerous tissue from non‐neoplastic HSG.^7^ In our case, we observed the hypoechoic areas under EUS, and furthermore, we could detect adenocarcinoma cells in the specimen obtained from the target area. This process suggested the application of NEWS as the diagnostic treatment.

NEWS allowed for full‐thickness resection of the gastric wall without a transmural opening. It is considered a minimally invasive endoluminal surgery for gastric SEL, and it may also be appropriate for lymph node‐negative early gastric cancer, which is difficult to remove by endoluminal therapy alone.^3^ Aoyama et al, summarized outcomes of NEWS for gastric SEL, and concluded that NEWS was characterized by an acceptable operative time, less blood loss, and fewer adverse events during the peri‐operative period. Also, NEWS contributed to no postoperative recurrence and preservation of gastric function during the follow‐up period.^8^

In our case, NEWS was selected as a diagnostic and therapeutic approach for the following reasons. First, in consideration of subsequent PD, a definitive diagnosis of the SEL by full‐thickness resection was required. Second, since adenocarcinoma was detected, the non‐exposure technique would prevent the peritoneal spread. Third, because the tumor measured approximately 30 mm in size, we presumed that the resected specimen could be removed per‐orally. Finally, since subsequent PD was scheduled in our case, removal of a smaller area of the stomach would be desirable.

During NEWS, lymph nodes were not dissected in our case, because a diagnosis of gastric cancer had not yet been established preoperatively, and PD was scheduled afterward. As a promising option for function‐preserving gastric surgery, local resection guided by sentinel node (SN) mapping has been developed. A multicenter randomized clinical trial showed the 5‐year disease‐free survival, disease‐specific survival, and overall survival was not different significantly between laparoscopic standard gastrectomy and laparoscopic SN navigation surgery.^9^ Furthermore, a combination of NEWS and SN basin dissection (SNBD) has been attempted in animals^10^ and in some clinical cases. Even though lymph nodes were not actually involved in our case, the combination of NEWS and SNBD might have been a choice.

In conclusion, our experience suggests that NEWS is a useful diagnostic and treatment option for gastric SEL, which is at risk of intraperitoneal dissemination by classic LECS.

## CONFLICT OF INTEREST STATEMENT

Takayuki Matsumoto is the responsible and executive JGES member for the DEN Open. The other authors declare no conflict of interest for this article.
